# Enjoy your journey: the bergamot polyphenols from the tree to the cell metabolism

**DOI:** 10.1186/s12967-021-03131-7

**Published:** 2021-11-04

**Authors:** Salvatore Nesci, Ernesto Palma, Vincenzo Mollace, Giovanni Romeo, Francesca Oppedisano

**Affiliations:** 1 Department of Veterinary Medical Sciences, University of Bologna, Ozzano Emilia, Italy; 2 Department of Health Sciences, Institute of Research for Food Safety & Health (IRC-FSH), University “Magna Graecia” of Catanzaro, Catanzaro, Italy; 3 Medical Genetics Unit, Sant’Orsola-Malpighi University Hospital, Bologna, Italy

**Keywords:** Bergamot, Cell metabolism, Flavonoids, Antioxidant, Dyslipidemia, Metabolic diseases

The Mediterranean-style diet was hypothesized by Ancel Keys who observed in Southern Italy the highest concentration of centenarians in the world associated with a low incidence of coronary heart disease, and a lower incidence of cancer and adult-onset diabetes mellitus overall [1]. Since 2010, the Mediterranean diet (Md) has been labelled as an “intangible cultural heritage of humanity” with the acknowledged benefits on longevity and life health-related quality. The Md highlights that unsaturated fatty acids, vitamins, and polyphenols, associated with constant physical activity, are compounded with beneficial effects on human health [1].

Bergamot (*Citrus bergamia Risso et Poiteau*) is an endemic plant growing in the Southern Italian region of Calabria, containing different classes of glycosylated flavonoids (e.g., flavanones and flavones) a group of polyphenolic natural substances, that can exert beneficial effects to prevent disease [2]. The microclimate and soil of Jonic Calabria confer specific organoleptic characteristics to the bergamot juice. Determining bergamot DNA sequence (≈ 300 Mbp) will have scientific importance for citrus taxonomy and phylogenetic tree reconstruction. Moreover, it will allow the development of a test for certification of bergamot fruit origin with obvious consequences for the defence of the authentic fruit grown in Calabria.

The bergamot polyphenolic fraction (BPF), a particular product derived from bergamot juice, contains flavonoids, furocoumarin and other polyphenols. The main flavanone compounds are naringenin, hesperetin, eriodictyol glycoside, while the main flavones present are apigenin, luteolin, chrysoerol, diosmetin. Bergamot flavonoids can counteract dyslipidemia and hyperglycemia. Moreover, bergamot juice-derived food supplements, enriched with pectins and vitamin C, can act on the adipose tissue feedback regulation and, according to the lipostat theory, significantly stimulate weight loss by increasing the levels of cardioprotective adiponectin, improve leptin and ghrelin levels, insulin sensitivity and reduce circulating insulin [3]. Since dyslipidemia is a well-established modifiable cardiovascular risk factor [4], the bergamot can have a homeostatic dose-dependent effect and potential synergistic action by statin administration on the human lipid profile. The main metabolism regulation effects are the total cholesterol decrease, triglycerides and low-density lipoprotein. Even if the bergamot supplementation molecular mechanism in dyslipidemia is not still understood, a possible explanation of cholesterol metabolism modulation is focused on its synthesis and faecal excretion. Therefore, the cellular targets are the 3-hydroxy-3-methyl-glutaryl-CoA (HMG-CoA) reductase, whose inhibition blocks the cholesterol endogenous synthesis, and the pancreatic cholesterol-ester-hydrolase and acyl-CoA cholesterol acyltransferase that are activated and, respectively, inhibited and fail esterification of endogenous cholesterol in human small intestinal mucosa by evading the cholesterol absorption [4].

In addition to the hypolipemic and hypoglycemic activities, the BPF guarantees antioxidant and anti-inflammatory effects [3]. Therefore, bergamot is effective in treating metabolic syndrome and has beneficial effects on the cardiovascular system. Consistently, an antioxidant response has been demonstrated both in vitro and in vivo. The effect leads to a reduction in cholesterol serum levels, glucose and triglycerides, a biological response accompanied by a reduction in systemic inflammation and, subsequently, an improvement in endothelial function. These effects were confirmed in patients treated with BPF, which showed a powerful effect in modulating the lipoproteins hepatic traffic and was able to counteract non-alcoholic liver disease (NAFLD). Specifically, brutieridin and melitidin have been shown to produce inhibition of HMG-CoA reductase. Recent data have also shown that BPF reduces cardiotoxicity induced by doxorubicin, counteracting the overproduction of reactive oxygen species (ROS), thus restoring protective autophagy and attenuating apoptosis and pathological myocardial remodelling [5]. Furthermore, mitochondrial dysfunction is the main event of ROS production in the cell, BPF promotes the correct coupling of the oxidative phosphorylation system and desensitizes the mitochondrial permeability transition pore formation, a phenomenon involved in triggering different forms of regulated cell death.

In addition, the BPF anti-inflammatory activity was tested in rats with cafeteria (CAF) diet-induced non-alcoholic steatohepatitis. The results obtained showed that BPF supplementation reduces the IL-6 expression levels and increases the anti-inflammatory protein IL-10 expression with the reduction in liver inflammation. Rats fed with CAF and treated with BPF show a reduction in inflammatory foci. Furthermore, the antioxidant and anti-inflammatory effects of a phytocomplex, called Bergacyn, made of bergamot polyphenols and cynaropicrin isolated from artichoke (*Cynara cardunculus*), on patients with type 2 diabetes and NAFLD improved health condition, accordingly the phytocomplex action on oxidative stress and inflammation. Indeed, there was an increase in glutathione peroxidase and superoxide dismutase levels, whereas malondialdehyde and TNF-α levels were reduced by contributing to better NO-mediated reactive vasodilation [3].

Bergamot contains a plethora of molecules. Therefore, it will be interesting, in the nutraceuticals field, to be able to identify the molecules protagonists in the disease’s prevention and the improvement of the cellular metabolism in pathological conditions. From a pharmacological point of view, it will be interesting to elucidate how the bergamot juice compounds act in a synergistic and/or additive way in carrying out their beneficial action on human health.

## Data Availability

Not applicable.
